# A Cohort Study of Traffic-Related Air Pollution and Mortality in Toronto, Ontario, Canada

**DOI:** 10.1289/ehp.11533

**Published:** 2009-01-05

**Authors:** Michael Jerrett, Murray M. Finkelstein, Jeffrey R. Brook, M. Altaf Arain, Palvos Kanaroglou, Dave M. Stieb, Nicolas L. Gilbert, Dave Verma, Norm Finkelstein, Kenneth R. Chapman, Malcolm R. Sears

**Affiliations:** 1 Division of Environmental Health Sciences, School of Public Health, University of California, Berkeley, Berkeley, California, USA;; 2 Department of Family and Community Medicine, Mount Sinai Hospital, University of Toronto, Toronto, Ontario, Canada;; 3 Meteorological Services, Environment Canada, Toronto, Ontario, Canada;; 4 School of Geography and Earth Science, McMaster University, Hamilton, Ontario, Canada;; 5 Healthy Environments and Consumer Safety Branch, Health Canada, Ottawa, Ontario, Canada;; 6 Program in Occupational Health & Environmental Medicine, McMaster University, Hamilton, Ontario, Canada;; 7 Division of Respirology, Department of Medicine, University of Toronto, Toronto, Ontario, Canada;; 8 Division of Respirology, Department of Medicine, McMaster University, Hamilton, Ontario, Canada

**Keywords:** air pollution, GIS, mortality, nitrogen dioxide, traffic air pollution, Toronto

## Abstract

**Background:**

Chronic exposure to traffic-related air pollution (TRAP) may contribute to premature mortality, but few studies to date have addressed this topic.

**Objectives:**

In this study we assessed the association between TRAP and mortality in Toronto, Ontario, Canada.

**Methods:**

We collected nitrogen dioxide samples over two seasons using duplicate two-sided Ogawa passive diffusion samplers at 143 locations across Toronto. We calibrated land use regressions to predict NO_2_ exposure on a fine scale within Toronto. We used interpolations to predict levels of particulate matter with aerodynamic diameter ≤ 2.5 μm (PM_2.5_) and ozone levels. We assigned predicted pollution exposures to 2,360 subjects from a respiratory clinic, and abstracted health data on these subjects from medical billings, lung function tests, and diagnoses by pulmonologists. We tracked mortality between 1992 and 2002. We used standard and multilevel Cox proportional hazard models to test associations between air pollution and mortality.

**Results:**

After controlling for age, sex, lung function, obesity, smoking, and neighborhood deprivation, we observed a 17% increase in all-cause mortality and a 40% increase in circulatory mortality from an exposure contrast across the interquartile range of 4 ppb NO_2_. We observed no significant associations with other pollutants.

**Conclusions:**

Exposure to TRAP was significantly associated with increased all-cause and circulatory mortality in this cohort. A high prevalence of cardiopulmonary disease in the cohort probably limits inference of the findings to populations with a substantial proportion of susceptible individuals.

Recent evidence suggests that chronic exposure to traffic-related air pollution (TRAP) may be associated with premature mortality. Dutch researchers demonstrated a near doubling of cardiopulmonary mortality for subjects living near major roads (Hoek et al. 2002), although a more recent follow-up with a larger sample produced generally smaller but still elevated risk estimates (Beelen et al. 2007). A study from Norway reported an increase of 18% in male all-cause mortality based on a comparison of the lowest to the highest quartile of exposure in nitrogen oxides estimated from a dispersion model (Nafstad et al. 2004). Canadian studies showed that subjects living close to major roads had mortality rate advancements of 2.5 years and a significant increase in all-cause mortality of 18% (Finkelstein et al. 2004). These large health effects might be attributable to higher intake fractions and subsequent doses for residents living near roadways (Marshall et al. 2005) or to the higher toxicity of traffic pollutants compared with other sources (Nel 2005; Schlesinger et al. 2006).

Studies of ambient particulate matter (PM) ≤2.5 μm in aerodynamic diameter (PM_2.5_) in California have shown that intraurban exposure gradients are associated with preclinical markers of atherosclerosis (Künzli et al. 2005) and risks of premature mortality (Jerrett et al. 2005). Using a sub-cohort of about 23,000 from the American Cancer Society cohort in Los Angeles, Jerrett et al. (2005) observed relative risks (RRs) three times greater than those reported in the national study (Pope et al. 2002), with the major difference being the within- rather than between-city exposure assignment. is increase in the risk may have been attributable to more accurate exposure assignment or the relatively higher contribution of traffic sources in Los Angeles compared with other cities in the United States.

These recent Los Angeles studies (Jerrett et al. 2005; Künzli et al. 2005) used geo-statistical interpolation models that capture regional patterns of pollution well, but may not fully account for near-source impacts from local traffic because of the limited variation in PM_2.5_ within cities and potential oversmoothing from the interpolation model used to assess exposure (Moore et al. 2007). The aforementioned European and Canadian studies used distance proxies for exposure to traffic, sparse government monitoring networks to interpolate background exposure, or dispersion models with limited cross- validation against measured data. To date, no study has used extensive field measurements with well-validated models to assess the association between TRAP and mortality. In this study, we applied 143 field measurements of nitrogen dioxide, an inorganic gas marker of TRAP, to a well-characterized cohort of patients from a respiratory clinic in Toronto, Ontario, Canada. Our objective was to assess the association between TRAP and mortality.

## Materials and Methods

### Study site description

We undertook our study in the City of Toronto, situated on the northern shore of Lake Ontario, Canada. Toronto is Canada’s largest city, with a metropolitan population of nearly 4.7 million in 2001 (Statistics Canada 2001). Several busy expressways and numerous urban arterial roads traverse the city, leading to a wide range of variation in traffic exposures and related pollutants.

### The study cohort

Subjects for this study were drawn from a respiratory disease clinic in the Toronto Western Hospital, University Health Network, for the purpose of studying the health effects of air pollution. Patients seen at the clinic were referred to a pulmonologist for investigation or management of a respiratory complaint. The study was approved by the research ethics boards of the Mt. Sinai Hospital, Toronto, and the St. Joseph’s Health Center, Hamilton.

All residents of Ontario are covered under the Ontario Health Insurance Plan (OHIP), a government-operated universal health insurance program. In 1992, unique personal health insurance numbers (HINs) were introduced to replace the previous insurance numbers, which were family based. We collected the HINs and other identifiers for all patients treated at the clinic between 1992 and the end of 1999.

Members of the cohort were linked to numerous administrative databases using the HIN as the identifier. These databases include the OHIP physician billing database, 1992–1999; the Ontario Hospital Discharge Database, 1992–1999; and the Ontario Mortality Registry, 1992–2002. In total, 2,414 patients with Toronto postal codes were identified. We extracted height, weight, results of lung function testing, and smoking history from the clinic’s database. Fifty-four subjects were missing data necessary to compute the body mass index (BMI). Because BMI is an important predictor of mortality, and potentially a major confounder, we chose to exclude subjects with missing BMI data, rather than to impute values.

Each claim for payment submitted to OHIP by physicians must include a diagnosis code. We used the OHIP linkage to classify underlying respiratory disease status by using diagnostic codes submitted to OHIP by specialist physicians. Diagnoses included chronic obstructive pulmonary disease (COPD) [*International Classification of Diseases, 9th Revision, Clinical Modification* (ICD-9-CM 2007) codes 491, 492, 496] and asthma (ICD-9 493). We classified subjects diagnosed with both asthma and COPD as having COPD (ICD-9-CM 2007). We also searched the OHIP billing file and Ontario hospital discharge database for diagnoses of diabetes (ICD-9-CM 250) and chronic ischemic heart disease (IHD) (ICD-9-CM 412–414). Subjects were classified with these disorders if the diagnosis had been made in two or more claims submissions by a general practitioner, one claim submission by a specialist, or in any hospitalization.

### Neighborhood contextual confounders

Evidence from social epidemiology suggests that neighborhood context may affect health independently beyond individual risk factors (Diez-Roux 2002). In Quebec, a deprivation index (DI) derived from the Canadian census was correlated with premature mortality due to tobacco-associated causes (Pampalon and Raymond 2000). Because a relationship may exist between neighborhood context and traffic exposures, we used a similar index to adjust mortality risks across the region of our study to control for confounding of the TRAP effect. We used data from the 1996 Census of Canada at the enumeration area (EA) level to derive a contextual variable. EAs are the smallest unit of aggregation in the Canadian census, with an average population of about 1,000. In a small number of cases (< 60), we assigned census tract or metropolitan area census data because EA data were suppressed by Statistics Canada to protect privacy because of small population counts in the EA. We employed principal components to generate the DI. Analysis showed that only the first principal component, accounting for 72% of the variance, was related to mortality. We thus retained only the first component, and we defined the DI as [−0.62 × log(mean income) + 0.58 × unemployment rate + 0.53 × proportion of residents who did not complete high school]. Higher scores on the DI indicate a less favorable combination of income, education, and employment.

### Exposure assessment

We collected NO_2_ samples using two-sided Ogawa passive diffusion samplers at 150 locations across Toronto. We selected sampling locations with a location-allocation model based on pollution variability over space and on residential population density summarized elsewhere (Kanaroglou et al. 2005). We measured NO_2_ levels across Toronto for two 2-week periods, one in early fall 2002 and the other in spring 2004. To assess the reliability of the samplers, we deployed two samplers in tandem at each location during the first round of monitoring, yielding a total of four separate samples at each location. After determining low variation between the duplicates from the 2002 monitoring, we deployed one single two-sided sampler at each location, but in about 15% of locations we used two two-sided samplers for the spring 2004 sampling. During each sampling period, we deployed 100 Ogawa samplers simultaneously. This deployment strategy eliminated the need for temporal adjustments to the measurements before developing land-use regression (LUR) models as has been necessary in other studies (Cyrys et al. 2005; Hochadel et al. 2006). During the second round, we kept 50 locations the same as those in the first round and added 50 new locations (a total 150 spatial locations and 143 valid measurements over the two sampling periods). We chose these new sites using the same location-allocation approach to improve spatial coverage of the model, whereas the 50 that were the same during both deployments provided an assessment of interseasonal correlation and hence the stability of the NO_2_ spatial pattern, which we subsequently used to assign exposure across the cohort. We sent the Ogawa filters to an Environment Canada laboratory for analysis, where NO_2_ levels were determined by ion chromatography (Jerrett et al. 2007).

We derived variables employed in subsequent LUR models as predictors of NO_2_ with the ArcGIS 9 geographic information system (GIS) software (ESRI, Redlands, CA, USA). LUR employs the measured pollutant levels as the dependent variable and an array of land use, traffic, physical geography, and population variables as predictors. The detailed modeling framework and results of the fall, first season of measurement are described by Jerrett et al. (2007), and the models used to predict exposures for the second, spring season are discussed by Finkelstein and Jerrett (2007). Briefly, we implemented a manual forward-selection procedure to select variables that explained the largest proportion of variation in NO_2_. We screened > 90 variables for each season, representing different potential predictors of exposure. In both models, *R*^2^ values were nearly 0.7. Each model contained variables representing roadways, population density, land use, and the geographic *x*-coordinate to represent a west-to-east trend of higher to lower NO_2_ across the city.

We also derived interpolated surfaces for PM_2.5_ and ozone. For the PM_2.5_ surface, we computed the annual geometric average for 2002 from 14 Ministry of the Environment of Ontario (MOE) stations, equipped with tapered element oscillating microbalance monitors (hourly measurements). This was the first year when a spatially extensive coverage of PM_2.5_ was available for the study area. For O_3_, we used 16 MOE sites and one Environment Canada site to generate annual averages. We computed annual averages for O_3_ for 1992, 1997, and 2002 (the beginning, middle, and end of the follow-up period). After interpolation, we averaged the three surfaces and assigned them to the subjects. The spatial domain of the samplers extended beyond our immediate study area to increase the stability of the interpolations, but the study area was approximately at the center of the interpolation surface. We performed interpolations using an inverse distance weighting algorithm in ArcGIS 9 (Burrough and McDonnell 1998).

### Statistical model

The analysis relied on Cox proportional hazards regression models run in STATA version 9.0 (StataCorp., College Station, TX, USA). We ran models for all nonaccidental causes, circulatory (ICD-9-CM 400–440), respiratory (ICD-9-CM 460–519), and lung cancer (ICD-9-CM 162) and, as a negative control, for all nonaccidental causes less circulatory, respiratory, and lung cancer deaths. For each outcome, we began with a model adjusted only for age and sex. We then added variables to control for confounding: clinical measures (BMI, lung function), smoking, and the DI. As a sensitivity analysis, we added as explanatory variables diagnoses of COPD, diabetes, and IHD, diseases probably on the causal pathway between pollution exposures and mortality, but also possibly representative of unmeasured confounders such as diet or occupational exposure. We adjusted SEs for clustering in census tracts. We tested deviations from the proportional hazards assumption by introducing interactions of time with all covariates.

## Results

Table 1 shows descriptive statistics for the cohort and the assigned exposures. In this sample, 48% were male, the median age was 60 years, and about 47% were current or former smokers.

**Figure 1:**
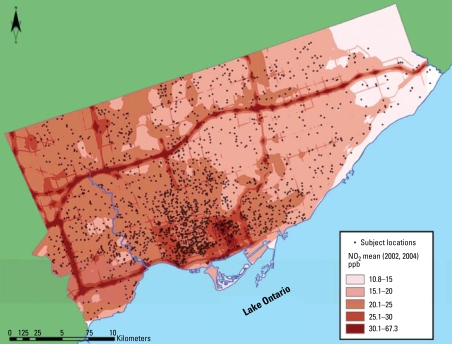
Average NO_2_ levels based on two LUR models calibrated from 2002 fall and 2004 spring monitoring campaigns with 143 monitors. Approximate subject coordinates are shifted to protect privacy.

**Figure 2:**
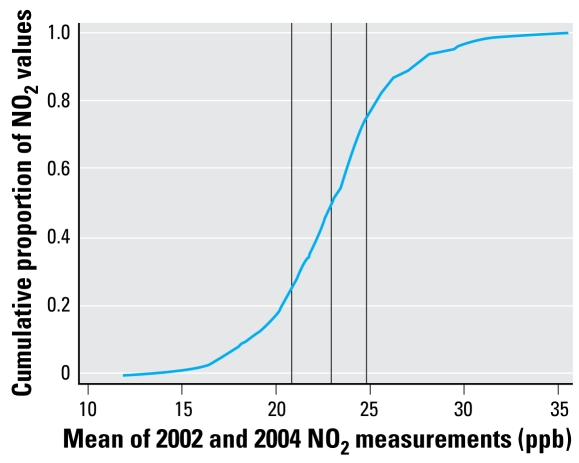
The cumulative distribution of mean NO_2_ exposures among study subjects. The vertical lines are the 25th, 50th, and 75th percentiles of the distribution.

Figure 1 presents a map showing the average of two main exposure models for NO_2_. Figure 2 shows the cumulative distribution of mean (2002 and 2004) NO_2_ exposures among the study subjects. Because of meteorologic variability, the magnitude of the 2-week average NO_2_ concentrations differed between seasons, and there were small differences in the predictors derived from LUR. However, the spatial patterns derived from the two season-specific LUR models were similar because the underlying pattern in NO_x_ emissions, influenced largely by the distribution of traffic, changes little over time. The prediction surfaces followed a similar spatial pattern of high concentrations in the downtown and around the major highways, with lower concentrations in the northeast of the city, where there is a zoo and relatively little human habitation or traffic. The two surfaces were highly correlated after assignment to the subject (Pearson’s *r* = 0.73), and were averaged for inclusion in the regression model [see Supplemental Material, Appendix, for more detail (http://www.ehponline.org/members/2009/11533/suppl.pdf)].

There were 10,500 person-years of follow-up in the cohort. Table 2 shows the results of the Cox survival models for the relations between interpolated NO_2_ and all-cause, circulatory, and respiratory mortality. The fourth column lists mortality from all nonaccidental causes except those in the first two columns. The first line of Table 2 shows models adjusted only for age and sex. The next line adds potential clinical confounders, including BMI and percent predicted forced vital capacity (FVC), expressed as a percentage of the normal value. We found no significant association between mortality and forced expiratory volume in the first second (FEV_1_) when FVC was included in the models. In the next line, we adjusted the models for smoking in three categories (i.e., never, ever, or current smoking) followed by adjustment for all the previous variables and the DI. Finally, as a sensitivity analysis, we added the chronic diseases COPD, IHD, and diabetes.

All-cause mortality was elevated in relation to the 4 ppb interquartile range (IQR) of NO_2_, with RRs ranging from 1.29 for the minimal adjustment model to 1.17 for the model with inclusion of all the control variables and adjustment for clustering within the census tract of residence. Confounding variables generally reduced the size of the NO_2_ effect, as expected. Including chronic diseases confounded the relationship mildly, lowering significance to slightly below conventional levels [hazard ratio (HR) = 1.15; 95% confidence interval (CI), 0.99–1.33].

Circulatory mortality was associated with NO_2_ in all models. In the model with maximal control for confounding, the RR significantly increased by about 40% over the IQR of NO_2_. We found minimal change in the effect estimate when we added the diagnoses of chronic conditions to the model or when we included the road buffer traffic marker (Table 3).

The point estimates for respiratory mortality were elevated, but the confidence intervals included unity. We attempted to model associations between lung cancer and NO_2_; however, there were only 35 deaths in the category, which resulted in positive estimates with very wide confidence intervals (results data not shown). The effect of NO_2_ exposure on other nonaccidental causes of death, minus cardio-pulmonary and lung cancer mortality, was confounded by the clinical and socioeconomic variables. In the full model, we found no significant association between these other non-accidental causes of death and NO_2_ exposures.

The hypothesis of proportionality in the hazards was not rejected for any model. We tested interactions between the explanatory variables and NO_2_, but none were significant.

In a neighboring city (Hamilton, Ontario), we found an association between residence locations close to traffic and an increased risk in all-cause and circulatory mortality (Finkelstein et al. 2004). We applied the same definition of traffic proximity (residence within 50 m of a major road or 100 m of a highway) to the Toronto cohort. Table 3 shows a large point estimate and a borderline significant association between residential proximity to traffic and circulatory mortality. In a model that included NO_2_, the effect of NO_2_ was significant and the risk attributable to the traffic marker was lower. We found no relation between proximity to traffic and other nonaccidental causes.

## Discussion

In this article we report the association between NO_2_, a marker for TRAP, and mortality in a cohort of subjects drawn from a pulmonology clinic in Toronto. TRAP was associated with significant elevations in mortality from all causes and from circulatory causes. With control for confounding, all-cause mortality remained elevated by 17% and RRs were nonsignificant only where adjusted for chronic diseases and the proximity to traffic exposure marker. Effect sizes over the IQR for all-cause mortality were similar to those reported in the Norwegian study (Nafstad et al. 2004).

Circulatory mortality remained significantly elevated with RRs in the range of 1.4 in models that controlled maximally for confounding and for neighborhood clustering on the census tracts. The HR for circulatory mortality was 2.28 over a 10-ppb contrast, which was present in the data. These effects are comparable in size to those reported in earlier European studies (Hoek et al. 2002) and are consistent with findings linking coronary artery calcification to distance from a major road (Hoffmann et al. 2007). More generally, effects on circulatory outcomes have been demonstrated to be larger than those for other mortality outcomes in relation to air pollution (Jerrett et al. 2005; Pope et al. 2004).

Respiratory mortality was also elevated in relation to NO_2_, although models only provided nonsignificant trends. Statistical power to detect the effects of NO_2_ on lung cancer mortality was limited because of a small number of deaths in this category, and the results were inconclusive. Nonaccidental deaths less circulatory, respiratory, and lung cancer causes were not associated with TRAP in models that controlled for major confounders.

In a previous study in Hamilton, Ontario (Finkelstein et al. 2004), we reported a relation between mortality and residence close to traffic, as indicated by a road buffer. In this study, when using the same traffic buffer, we found a nonsignificant effect of about the same size as in the earlier Hamilton study. In a model that included both the traffic buffer indicator variable and NO_2_, the effect of NO_2_ remained significant, whereas the effect of the traffic indicator was reduced. Mean values of NO_2_ were about 22 ppb outside the buffer and about 25 ppb inside the buffer. This suggests that the traffic indicator is a surrogate for NO_2_ and possibly other near-source traffic-related exposures. Other constituents likely to be higher by roadways include carbon monoxide, ultrafine PM, volatile organic compounds, and elemental carbon (Reponen et al. 2003; Zhu et al. 2002). A recent study from Toronto suggests that the highest gradient in near-road exposure is in ultrafine PM counts (Beckerman et al. 2008).

We examined the relation between other exposure variables and mortality in our cohort. Exposure contrasts for PM_2.5_ and O_3_ were small in Toronto (IQR of about 1 μg/m^3^ or 1 ppb, respectively), and we found no significant associations with mortality in any of the models (data not shown). The combination of a relatively small number of deaths in the cohort and a limited exposure contrast limited the statistical power to detect mortality effects. Consequently, no definitive conclusions can be drawn about these associations with PM_2.5_ and O_3_.

One unique strength of this study was our ability to control for clinically ascertained preexisting conditions and lung function. These data allowed us to apply controls based only on self-reports in other studies of chronic mortality (e.g., Hoek et al. 2002; Jerrett et al. 2005; Pope et al. 2002). We included preexisting diseases as a sensitivity analysis. Including these conditions may have overcontrolled for confounding because some of the variables may be on the causal pathway from pollution to mortality. For example, preclinical markers of IHD have been associated with TRAP or PM partly arising from traffic (Künzli et al. 2005). There is also some indication that COPD may associate with TRAP (Schikowski et al. 2005) and with diabetes (Brook et al. 2008). In such instances, inclusion of the preexisting conditions would remove some of the true effect attributable to air pollution. Even with this potential for overcontrol, we found only a small impact on the risk estimates for NO_2_ on all-cause and circulatory mortality. We subsequently addressed effect modification by disease status, but we found no significant effect modifiers identified. Although this cohort is enhanced with individuals with preexisting conditions, the results are generally insensitive to inclusion of these conditions as either confounders or effect modifiers. One explanation for the lack of influence of chronic disease status as a confounder or modifier may arise from the role of air pollution in disease formation. All of the chronic conditions tested were independently and positively associated with mortality in this cohort. If air pollution did contribute to disease formation, and these diseases were associated with elevated mortality, these disease states may not modify or confound the association between mortality and air pollution. In this case, the effects of air pollution would be represented in elevated mortality related to chronic disease status. This cohort lacked the statistical power to use structural equation models to assess the pathway model directly, but a larger cohort is currently under construction that will allow for structural models to address this hypothesis.

The exposure assessment used in this study relied on extensive field measurements and models capable of predicting fine-scale variation in TRAP. Few other studies have used such an extensive network of field measurements to characterize the likely ambient exposures. Although we took the measurements over short periods, the spatial pattern appears relatively stable over time. For the 43 sites where the NO_2_ was available in both rounds of measurement, the correlation coefficient *r*-value was 0.82, and both seasons had a similar spatial structure [see Supplemental Material, Appendix, for details (http://www.ehponline.org/members/2009/11533/suppl.pdf)]. The relative stability over time of the spatial pattern of pollutant concentrations derived from short-term saturation monitoring has been reported by others (Lebret et al. 2000; Sahsuvaroglu et al. 2006). Thus, although the measurement period for NO_2_ was at or beyond the end of mortality follow-up, there is evidence suggesting spatial exposure contrast observed from these shorter periods captures the essential aspects of the chronic exposure experience for the cohort. With the accurate predictions from the LUR models, the assigned exposures appear to reduce measurement error, which may have contributed to the large effect size for circulatory mortality. The road distance buffers tested here and used in comparable studies (Finkelstein et al. 2004) did produce elevated risks, but with less certainty than with the predicted NO_2_ concentrations, as would be expected from a less precise exposure estimator (Molitor et al. 2007). The exposure assessment appears to diminish measurement error and produce more certain estimates, but we are unable to comment directly on which specific constituents of the pollutant mixture are linked to the health effects. Research efforts are under way to measure speciated PM and volatile organic compounds to better understand what constituents of the traffic mixture are most closely related to the observed risks.

We based NO_2_ estimates on two simultaneous 2-week monitoring periods. This method obviates the need to account for temporal effects in different measurements, but for most of the year we had no monitoring at these locations. This approach may have resulted in invalid estimates of the annual average. To assess this possibility, we compiled 2-week averages for the downtown Toronto central monitoring site operated by the Ontario MOE. We compared the 2-week averages with the annual averages based on daily data. The annual average was 23.2 ppb, with a minimum of 18.9 ppb and maximum of 30.9 ppb. The mean difference in absolute values between the 2-week average and the annual average is 2.39 ppb, with a maximum difference of 7.72 ppb and a minimum difference of 0.014 ppb. On average, then, we found about a 10% difference between the annual mean and the 2-week averages. In an earlier publication documenting the exposure modeling (Jerrett et al. 2007), we compared the 2-week average values to the monthly average and 5-year averages at the three government monitors where co-located measurements were available. The NO_2_ levels were within 4.1–27.7% of the 5-year average. These analyses suggest that the two simultaneous 2-week samples probably reflect the annual and 5-year average values well. Similar studies in Southern California indicated high interclass correlations > 0.9 between the annual mean and two or three 2-week average measurements of NO_2_ (Gilliland et al. 2005).

Other risk factors near roadways may also have contributed to the elevated mortality. Traffic noise is a particular concern given similar source contributions compared with NO_2_ and the previous research linking traffic noise to elevated myocardial infarctions (Babisch et al. 2005). In this study, we are unable to rule out confounding by noise, and there may be residual influence from this unmeasured risk factor. Studies combining noise and air pollution exposure are a priority for future research.

Because we used existing clinic data rather than prospectively collected information, we did not obtain some valuable information on individual risk factors. For example, socioeconomic status was not available at the individual level, although neighborhood data did appear to be a useful surrogate. There is thus the possibility of residual confounding by individual factors that could not be measured with this study design.

Although this study benefited from a well-characterized clinical cohort, generalizability of the results could be questioned given the high prevalence of chronic diseases relative to the general population: Nearly half the subjects had been diagnosed with IHD, and 30% had COPD. The high prevalence of cardio-pulmonary disease in the cohort probably limits inference of the findings to populations with a substantial proportion of susceptible individuals. The results must also be viewed with the caveat that the sample size was smaller than in other cohort studies of mortality, and with small samples there is a higher possibility of chance findings. In follow-up research, a larger more representative sample of patients from general-practice clinics will be investigated.

The reported findings may be influenced by the inclusion of susceptible individuals, but the large risks uncovered here and in the few other studies focused on traffic implicate TRAP as a risk to public health. When viewed in the context of increasing miles traveled (Frumkin et al. 2004) and the rapid rise in automobile sales in newly industrialized nations such as China and India (Molina and Molina 2004), the health effects observed here may have growing relevance in relation to the prevention of disease and premature mortality.

## Figures and Tables

**Table 1:** Characteristics of subjects and their neighborhoods

Characteristic	Measure
No. of subjects	2,360
Males	1,128 (48)
Age	49, 60, 69
Smoking	
Never	1,248 (53)
Former	842 (36)
Current	270 (11)
FVC (% predicted)	77, 93, 105
FEV_1_ (% predicted)	61, 82, 99
BMI	23.8, 27.3, 31.5
Diagnosed with asthma	462 (20)
Diagnosed with COPD	698 (30)
Diagnosed with IHD	1,129 (48)
Diagnosed with diabetes	412 (17)
Median household income (CD$1,000)	33, 39, 50
Residence within 50 m of major road or 100 m from highway	570 (24)
Interpolated NO_2_ (ppb) (average of 2002 and 2004 sampling)	20.8, 22.9, 24.8
Interpolated PM_2.5_ (μg/m^3^)	8.64, 8.71, 8.83
Interpolated O_3_ (ppb)	17.5, 18.3, 18.8
Nonaccidental deaths (no.)	299
Circulatory deaths (ICD-9-CM codes 400–449) (no.)	82
Respiratory deaths (ICD-9-CM codes 460–519) (no.)	75

Values are no. (%) or 25th percentile, median, and 75th percentile.

**Table 2:** RR (95% CI) for mortality

Model	All nonaccidental causes (*n* = 298)	Circulatory (*n* = 80)	Respiratory (*n* = 75)	All nonaccidental causes of death less circulatory, respiratory, and lung cancer (*n* = 109)
Baseline: age, sex, mean NO_2_	1.29 (1.13–1.48)	1.52 (1.19–1.95)	1.11 (0.84–1.47)	1.23 (0.98–1.53)
+ BMI, BMI^2^, FVC (% predicted)	1.24 (1.08–1.42)	1.47 (1.14–1.90)	1.10 (0.83–1.45)	1.17 (0.94–1.47)
+ Smoking (current, former, never)	1.21 (1.06–1.39)	1.44 (1.11–1.86)	1.10 (0.83–1.46)	1.12 (0.89–1.40)
+ DI	1.17 (1.01–1.35)	1.45 (1.11–1.91)	1.06 (0.79–1.43)	1.06 (0.83–1.35)
Final model clustered on census tract	1.17 (1.00–1.36)	1.45 (1.10–1.92)	1.06 (0.67–1.49)	1.06 (0.84–1.34)
Sensitivity analysis: add diagnoses of COPD, chronic IHD, and diabetes	1.15 (0.99–1.33)	1.40 (1.05–1.86)	1.09 (0.78–1.54)	1.05 (0.83–1.33)

Data are the adjusted RR per IQR increase in mean NO_2_ (2002 and 2004). The first line is the baseline model (age, sex, NO_2_), and the subsequent lines give the adjusted RR as additional confounders are added to the baseline model.

**Table 3:** RR (95% CI) for mortality in relation to residential proximity to traffic and an IQR increase in mean NO_2_ (2002 and 2004)

Model	All nonaccidental causes of death (*n* = 298)	Circulatory (*n* = 80)	All nonaccidental causes of death less circulatory, respiratory, and lung cancer (*n* = 109)
Model 1: traffic marker is the exposure variable	1.19 (0.92–1.53)	1.48 (0.91–2.42)	1.17 (0.74–1.84)
Model 2: traffic marker + NO_2_			
Traffic marker	1.11 (0.85–1.45)	1.22 (0.74–2.02)	1.15 (0.71–1.85)
NO_2_	1.13 (0.97–1.32)	1.39 (1.05–1.85)	1.03 (0.81–1.31)

Each model was adjusted for all of the confounders controlled for in the final model from Table 2. The first result is for the model in which proximity to traffic is the only exposure variable. The second result is for models in which both the traffic marker and NO_2_ are included together.
